# Budd-Chiari Syndrome Secondary to Myelofibrosis in a Patient With Polycythemia Vera: A 16-Year Disease Progression Case Highlighting JAK2 Mutation Pathogenesis

**DOI:** 10.7759/cureus.82629

**Published:** 2025-04-20

**Authors:** Huyu Jiao, Zhengang Zhang

**Affiliations:** 1 Internal Medicine, Tongji Hospital, Tongji Medical College, Huazhong University of Science and Technology, China, Wuhan, CHN; 2 Gastroenterology, Tongji Hospital, Tongji Medical College, Huazhong University of Science and Technology, China, Wuhan, CHN

**Keywords:** budd-chiari syndrome, jak2/stat gene, myelofibrosis, myeloproliferative neoplasms, polycythemia vera

## Abstract

Budd-Chiari syndrome (BCS) is a rare, life-threatening condition often caused by myeloproliferative neoplasms (MPNs), with polycythemia vera (PV) and essential thrombocythemia being common culprits. Myelofibrosis (MF)-related BCS is rare. We report a 45-year-old male patient with a 16-year history of PV that progressed to MF in 2021 and then to BCS in December 2022. The patient presented with abdominal distension and hepatomegaly, and imaging confirmed hepatic venous outflow obstruction. Treatment included anticoagulation, diuretics, and transjugular intrahepatic portosystemic shunt. This case highlights the delayed progression from PV to MF and subsequent BCS and the thrombotic risks in advanced MPN subtypes.

## Introduction

Budd-Chiari syndrome (BCS) is a rare yet potentially life-threatening vascular disorder characterized by obstructive lesions occurring anywhere from the hepatic veins to the junction of the inferior vena cava (IVC) and right atrium. Based on etiological classifications, two distinct subtypes are recognized: primary BCS, predominantly caused by intravascular thrombosis, and secondary BCS, resulting from extrinsic compression or neoplastic vascular invasion [1]. Epidemiologically, the syndrome demonstrates an estimated annual incidence of one case per 110,000 individuals and a population prevalence of two cases per million, underscoring its rarity yet significant clinical impact when encountered [2,3]. Studies have shown that myeloproliferative neoplasms (MPNs) are the most common underlying disease leading to BCS and almost half of the cases of BCS are associated with MPNs [4]. The most common subtype of BCS secondary to MPNs is polycythemia vera (PV), followed by primary essential thrombocythemia (ET) [5]. In contrast, BCS induced by myelofibrosis (MF) is less common. This report presents an exceptional case of a 45-year-old male patient initially diagnosed with PV 16 years ago, who subsequently progressed to MF in 2021 and was further diagnosed with BCS in December 2022 at our institution. This case underscores the potential association between MF and BCS and highlights the importance of exploring therapeutic strategies tailored to this unique clinical scenario. Early screening for JAK2 mutations in MPN patients facilitates risk stratification, enabling the implementation of prophylactic interventions aimed at reducing the incidence of BCS development.

## Case presentation

**Figure 1:**
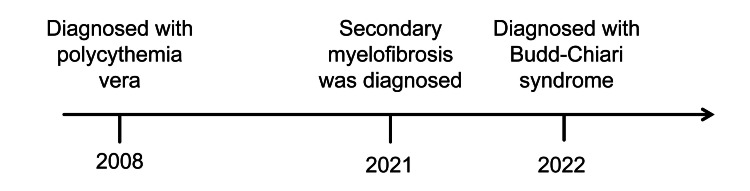
Disease progression timeline chart.

This case involves a 45-year-old male patient who was admitted to the hospital with bilateral lower extremity edema and abdominal distension that had persisted for more than a year, accompanied by symptoms of black stools for almost three days. During the course of his illness, the patient also experienced six months of malaise, loss of appetite, anorexia, diarrhea (up to 10 times per day with unformed stools), and discomfort such as panic and chest tightness. The patient's past medical history included PV and MF (Figure 1), but he denied having chronic diseases such as diabetes mellitus and coronary artery disease, and he was not infected with hepatitis B, tuberculosis, or other infectious diseases.

A thorough examination of the patient's longitudinal medical records revealed an initial presentation of splenomegaly identified incidentally in 2008 during a hospital visit. Unfortunately, the original imaging and laboratory reports from this period are no longer retrievable due to archival data loss. Subsequent hematologic surveillance between 2012 and 2023 (Table 1), as visualized in the provided temporal graph, demonstrated a progressive decline in erythrocyte count and hemoglobin concentration paralleling disease advancement. In contrast, leukocyte and platelet indices exhibited minimal fluctuation during this interval, maintaining relatively stable trajectories. Throughout the entire observation period, coagulation-related parameters, particularly D-dimer levels, were mostly elevated above the normal reference range, while hepatic function indices consistently remained within the normal reference interval. Comprehensive thrombophilia evaluation was performed to investigate potential hereditary predispositions to thrombotic complications. This included molecular diagnostic testing for factor V Leiden mutation, the G20210A mutation of the thrombospondin gene. All genetic analyses returned negative findings, indicating absence of these common hereditary thrombophilic variants in the patient's genotype.

**Table 1:** Previous laboratory test results. HB: hemoglobin; WBC: white blood cell; PLT: platelet; ALT: alanine aminotransferase; AST: aspartate aminotransferase.

Detection time	HB (reference range: 130-175g/L)	WBC (reference range: 3.5-9.5×10⁹/L)	PLT (reference range: 125-350×10⁹/L)	D-dimer (reference range: 0-0.5ug/mL)	ALT (reference range: 9-50U/L)	AST (reference range: 15-40U/L)
September 04, 2012	182	14.77	372	0.56	＜4	14
March 12, 2013	182	14.81	416	0.34	8	29
May 31, 2013	188	13.66	266	0.97	＜4	16
September 25, 2013	189	14.69	322	0.23	＜4	16
January 04, 2014	204	21.5	562	0.43	5	17
April 02, 2014	219	18.93	417	0.16	6	13
March 16, 2015	186	9.94	451	1.2	＜4	14
October 16, 2015	204	19.16	417	0.73	＜4	11
May 25, 2016	154	8.43	307	0.81	＜4	12
June 13, 2017	204	14.55	391	0.27	8	17
May 16, 2018	187	8.54	275	0.78	＜4	23
December 04, 2018	164	5.21	330	0.49	11	12
August 31, 2019	165	10.06	485	1.05	＜4	11
November 11, 2020	186	25.71	480	1.57	＜4	10
November 23, 2021	126	10.25	338	1.41	＜4	8
March 02, 2022	110	5.22	229	1.69	6	14
November 22, 2022	112	19.01	516	1.23	＜4	19
December 21, 2022	107	8.95	250	1.31	＜4	18
July 04, 2023	85	25.35	663	1.49	＜4	13
September 19, 2023	51	9.95	495	1.32	＜4	7
December 05, 2023	64	18.39	578	1.44	＜4	9
December 18, 2023	64	12.84	454	1.05	＜4	10

**Figure 2:**
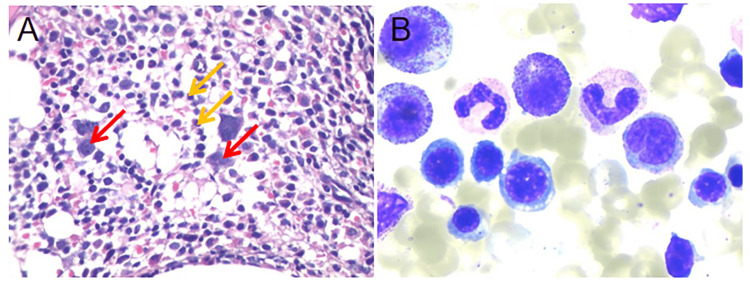
Bone marrow biopsy supports the diagnosis of secondary myelofibrosis: (A) Bone marrow biopsy shows active proliferation of granulocytes (yellow arrows) and megakaryocytes (red arrows) and (B) Bone marrow cytomorphology is suggestive of active bone marrow proliferation.

**Figure 3:**
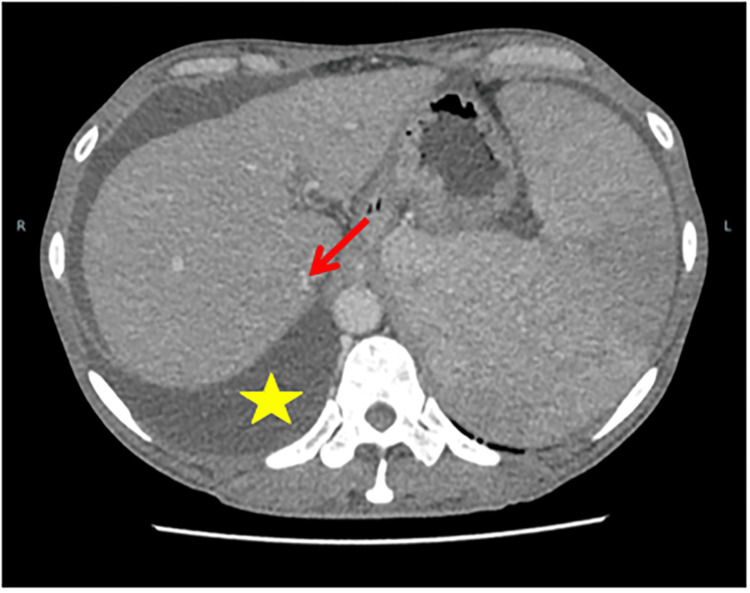
Abdominal CT: The liver was enlarged in size with splenomegaly, the hepatic segment of the inferior vena cava was obviously compressed (shown by the red arrow), and speckled enhancement was seen near the porta hepatis. A large amount of ascites was seen (yellow pentagram).

Through generation one and generation two sequencing, we found that the patient was positive for the JAK2 mutation. Combined with bone marrow cytology and biopsy results, the patient was then comprehensively diagnosed with PV. The main treatments included cytoreductive therapy with oral hydroxyurea, bloodletting and erythrocyte removal, and although the treatment was moderately effective, the patient's condition was recurrent. At a subsequent follow-up examination, a bone marrow cytology in 2021 revealed that the patient had developed secondary myelofibrosis (SMF) (Figure 2). Abdominal CT scan results on this admission showed significant compression of the hepatic segment of the IVC (Figure 3). Combining the patient's history information and the results of various examinations, we finally diagnosed the patient with MF caused by PV, which led to BCS. A revascularization strategy was adopted to address the patient's condition. Using the Seldinger technique, we successfully punctured the patient's right femoral vein and placed a 5F catheter sheath. Subsequently, the catheter was precisely placed into the IVC and an intrahepatic venography was performed. The imaging results showed that the hepatic segment of the IVC was slightly slender. A balloon dilatation of the IVC was performed and the patient was discharged from the hospital in improved condition. After discharge, the patient continued to receive anticoagulation therapy, and the symptoms of ascites and lower limb edema were significantly relieved.

## Discussion

MF is a chronic proliferative condition [6], which belongs to the category of BCR-ABL1-negative clonal stem cell disorders, whose main manifestations include ineffective erythropoiesis, dysplastic hyperplasia of megakaryocytes, and an increase in the proportion of immature granulocytes [7]. The pathogenesis of the disease involves not only clonal proliferation of stem cells but also an increase in the number of stromal cells, an increase in angiogenesis, and the osteosclerotic process. Among the known mutations that trigger MF, mutations in the JAK2, CALR, and MPL genes predominate, and these are directly and closely associated with the pathogenesis of the disease through activation of the JAK2/STAT signaling pathway [8].

Of note, approximately 15% of patients with essential ET or PV will progress to MF during the course of the disease, a phenomenon known as SMF. The progression of PV and ET to MF is part of the natural evolution of the disease, with a median time from the diagnosis of PV or ET to the development of MF of 8-16 years and 8.5 -13 years [9]. The WHO criteria for diagnosing MF integrate bone marrow biopsy, genetics, and clinical features. They require megakaryocyte changes with fibrosis, exclusion of other myeloid diseases, and specific mutations (JAK2, MPL, CALR). Diagnosis often needs three major criteria or two major plus two minor (e.g., anemia, splenomegaly). Staging depends on fibrosis severity, and other conditions like secondary MF must be ruled out [10].

For patients progressing to MF, international guidelines continue to recommend JAK inhibitors as the preferred first-line therapy for thromboprophylaxis [11]. JAK inhibitors, exemplified by ruxolitinib, exert their effects through specific blockade of the JAK-STAT signaling pathway, effectively suppressing the release of pro-inflammatory cytokines such as interleukin-6 (IL-6) and tumor necrosis factor-α, while significantly reducing microparticle-associated procoagulant activity. This multi-target synergistic mechanism disrupts the pathological basis of thrombosis. Clinical studies have demonstrated that ruxolitinib treatment significantly reduces thrombotic risk in MF patients, with post-treatment microparticle activity showing no significant difference compared to healthy controls [12]. Pre-treatment evaluation requires a comprehensive assessment, including complete blood count (CBC), hepatic and renal function tests, JAK2/CALR/MPL mutational profiling, and cardiovascular risk stratification. The therapeutic monitoring protocol adopts a two-phase approach: during the initial treatment phase, CBC, inflammatory markers (IL-6, C-reactive protein), and symptom scores (MPN-10 scale) are monitored every 4-8 weeks; in the long-term management phase, CBC, splenomegaly index, and bone marrow biopsy are reviewed every three months, with cardiovascular risk reassessment updated every six months [11,13]. 

BCS is an extremely rare disease worldwide. The common cause of BCS is closely related to blood being in a hypercoagulable state, which can be hereditary or acquired [14]. Inherited hypercoagulable states mainly include coagulation factor V Leiden mutation, protein C deficiency, and protein S deficiency. Acquired hypercoagulable states, on the other hand, are more commonly associated with myeloproliferative disorders, which account for more than half of BCS patients. PV accounts for 10% to 40% of reported cases, while primary ET and MF are relatively less common etiologies [3]. The clinical presentation of BCS lacks specificity, and patients' symptoms can range from completely asymptomatic to acute severe liver failure, which leads to more common misdiagnoses and missed diagnoses. The prognosis of the disease is usually poor, with 80% of patients unfortunately dying within three years of diagnosis [15].

To address the treatment options for BCS, we synthesized the 2021 APASL guideline [16] and concluded that anticoagulation is established as the first-line treatment option, and all patients with BCS should receive this treatment to reduce the risk of thrombus extension and new thrombus. Initially, patients are advised to take oral low molecular weight heparin for five to seven days, followed by a switch to lifelong anticoagulation with warfarin, with the goal of maintaining an international normalized ratio in the range of two to three. In patients with concomitant MPN, antiproliferative therapy with alpha interferon or hydroxyurea is required in addition to anticoagulation. In particular, patients with PV should have a hematocrit controlled below 45% [17].

When a patient develops a thrombus, the involved hepatic vein can be treated by injection of thrombolytic agents or mechanical thrombolysis using a balloon catheter. It has been shown that endovascular intervention is the preferred first-line treatment for selected BCS patients, and that this treatment is safe and effective, providing long-term survival for BCS patients [18]. For patients with poor thrombolysis and ineffective angioplasty recanalization, derivative techniques may be considered for treatment, including transjugular intrahepatic portosystemic shunt (TIPS) and surgical shunt. Compared with liver transplantation, TIPS has the advantages of less harm to patients, significant efficacy, and low postoperative morbidity, making it the main choice of derivative treatment.

If all of the above treatments are ineffective, liver transplantation may be considered. The five-year survival rate of BCS patients undergoing liver transplantation is approximately 75% [19]. In patients after liver transplantation, long-term anticoagulation therapy is still required to prevent the formation of new thrombi [20].

Our case provides novel insights into the temporal relationship between JAK2-driven clonal evolution and thrombotic complications. The 14-year latency from PV diagnosis to BCS manifestation aligns with the proposed "second hit" hypothesis, where chronic JAK/STAT activation induces endothelial dysfunction through: (i) increased circulating prothrombotic microparticles, (ii) upregulated P-selectin expression, and (iii) IL-6-mediated acute-phase response [17,20]. Notably, the patient's D-dimer elevation despite therapeutic anticoagulation suggests ongoing subclinical thrombosis, a hallmark of MPN-associated hypercoagulability. Therefore, patients with a history of myeloproliferative syndrome should be highly alert to the possibility of BCS in the presence of abdominal pain, ascites, and hepatomegaly.

## Conclusions

This case highlights the critical association between JAK2V617F-driven MF and BCS in a patient with 16-year PV progression. Successful endovascular recanalization and tailored anticoagulation achieved sustained remission, emphasizing the viability of early intervention in MPN-related thrombosis. The persistent JAK2 mutation underscores its dual role in fibrotic transformation and endothelial hypercoagulability. Prophylactic JAK inhibition and routine splanchnic vein screening should be considered for high-risk MF patients to mitigate life-threatening thrombotic complications, warranting multidisciplinary collaboration in MPN management.

## References

[1] Ludwig J, Hashimoto E, McGill DB, van Heerden JA (1990). Classification of hepatic venous outflow obstruction: ambiguous terminology of the Budd-Chiari syndrome. Mayo Clin Proc.

[2] (2018). EASL Clinical Practice Guidelines: management of hepatocellular carcinoma. J Hepatol.

[3] Li Y, De Stefano V, Li H, Zheng K, Bai Z, Guo X, Qi X (2019). Epidemiology of Budd-Chiari syndrome: a systematic review and meta-analysis. Clin Res Hepatol Gastroenterol.

[4] Smalberg JH, Arends LR, Valla DC, Kiladjian JJ, Janssen HL, Leebeek FW (2012). Myeloproliferative neoplasms in Budd-Chiari syndrome and portal vein thrombosis: a meta-analysis. Blood.

[5] Seijo S, Plessier A, Hoekstra J (2013). Good long-term outcome of Budd-Chiari syndrome with a step-wise management. Hepatology.

[6] Neuwirtová R, Mociková K, Musilová J, Jelínek J, Havlíček F, Michalová K, Adamkov M (1996). Mixed myelodysplastic and myeloproliferative syndromes. Leuk Res.

[7] Tefferi A (2000). Myelofibrosis with myeloid metaplasia. N Engl J Med.

[8] Arber DA, Orazi A, Hasserjian RP (2022). International Consensus Classification of Myeloid Neoplasms and Acute Leukemias: integrating morphologic, clinical, and genomic data. Blood.

[9] Cerquozzi S, Tefferi A (2015). Blast transformation and fibrotic progression in polycythemia vera and essential thrombocythemia: a literature review of incidence and risk factors. Blood Cancer J.

[10] Khoury JD, Solary E, Abla O (2022). The 5th edition of the World Health Organization Classification of Haematolymphoid Tumours: Myeloid and Histiocytic/Dendritic Neoplasms. Leukemia.

[11] Tefferi A (2023). Primary myelofibrosis: 2023 update on diagnosis, risk-stratification, and management. Am J Hematol.

[12] Verstovsek S, Mesa RA, Livingston RA, Hu W, Mascarenhas J (2023). Ten years of treatment with ruxolitinib for myelofibrosis: a review of safety. J Hematol Oncol.

[13] Thiele J, Kvasnicka HM, Gianelli U (2025). Evolution of WHO diagnostic criteria in "Classical Myeloproliferative Neoplasms" compared with the International Consensus Classification. Blood Cancer J.

[14] Menon KV, Shah V, Kamath PS (2004). The Budd-Chiari syndrome. N Engl J Med.

[15] Garcia-Pagán JC, Valla DC (2023). Primary Budd-Chiari syndrome. N Engl J Med.

[16] Shukla A, Shreshtha A, Mukund A (2021). Budd-Chiari syndrome: consensus guidance of the Asian Pacific Association for the study of the liver (APASL). Hepatol Int.

[17] Marchioli R, Finazzi G, Specchia G (2013). Cardiovascular events and intensity of treatment in polycythemia vera. N Engl J Med.

[18] Mukhiya G, Zhou X, Han X, Jiao D, Pokhrel G, Li Y, Pokhrel S (2022). Evaluation of outcome from endovascular therapy for Budd-Chiari syndrome: a systematic review and meta-analysis. Sci Rep.

[19] de Franchis R (2005). Evolving consensus in portal hypertension. Report of the Baveno IV consensus workshop on methodology of diagnosis and therapy in portal hypertension. J Hepatol.

[20] Janssen HLA, Garcia-Pagan JC, Elias E (2003). Budd-Chiari syndrome: a review by an expert panel. J Hepatol.

